# The effect of preoperative chemoradiotherapy on lymph nodes harvested in TME for rectal cancer

**DOI:** 10.1186/1477-7819-11-292

**Published:** 2013-11-18

**Authors:** Stefano Scabini, Fabrizio Montecucco, Alessio Nencioni, Gabriele Zoppoli, Marina Sartini, Edoardo Rimini, Andrea Massobrio, Luisito De Marini, Alessandro Poggi, Roberto Boaretto, Emanuele Romairone, Alberto Ballestrero, Valter Ferrando

**Affiliations:** 1 Oncologic Surgery and Implantable Systems Unit, Department of Emergency, IRCCS San Martino IST, Genoa, Italy; 2 Salita della Madonnetta 20/10, 16136 Genoa, Italy; 3 First Clinic of Internal Medicine, Department of Internal Medicine, University of Genoa School of Medicine. IRCCS Azienda Ospedaliera Universitaria San Martino–IST Istituto Nazionale per la Ricerca sul Cancro, Genoa, Italy; 4 Oncologic Unit, Department of Internal Medicine (DiMI), University of Genoa, Genoa, Italy; 5 Department of Health Sciences, University of Genoa, Genoa, Italy; 6 Oncologic Molecular and Angiogenesis Unit, IRCCS San Martino IST, Genoa, Italy

**Keywords:** Chemoradiation, Lymph nodes, Neoadjuvant, Rectal cancer

## Abstract

**Background:**

Adequate lymph nodes resection in rectal cancer is important for staging and local control. This retrospective analysis single center study evaluated the effect of neoadjuvant chemoradiation on the number of lymph nodes in rectal carcinoma, considering some clinicopathological parameters.

**Methods:**

A total of 111 patients undergone total mesorectal excision for rectal adenocarcinoma from July 2005 to May 2012 in our center were included. No patient underwent any prior pelvic surgery or radiotherapy. Chemoradiotherapy was indicated in patients with rectal cancer stage II or III before chemoradiation.

**Results:**

One-hundred and eleven patients were considered. The mean age was 67.6 yrs (range 36 – 84, SD 10.8). Fifty (45.0%) received neoadjuvant therapy before resection. The mean number of removed lymph nodes was 13.6 (range 0–39, SD 7.3). In the patients who received neoadjuvant therapy the number of nodes detected was lower (11.5, SD 6.5 vs. 15.3, SD 7.5, p = 0.006). 37.4% of patients with preoperative chemoradiotherapy had 12 or more lymph nodes in the specimen compared to the 63.6% of those who had surgery at the first step (p: 0.006).

Other factors associated in univariate analysis with lower lymph nodes yield included stage (p 0.005) and grade (p 0.0003) of the tumour. Age, sex, tumor site, type of operation, surgeons and pathologists did not weight upon the number of the removed lymph nodes.

**Conclusion:**

In TME surgery for rectal cancer, preoperative CRT results into a reduction of lymph nodes yield in univariate analisys and linear regression.

## Background

The gold standard for rectal cancer is the total mesorectal excision (TME) who allow adequate resection of the tumour and the regional lymph nodes. The International Union Against Cancer (UICC) and the American Joint Committee on Cancer (AJCC) decided to recommend a minimum of 12 lymph nodes for an adequate staging of colorectal cancer [1] in accordance with the guideline of the World Congress of Gastroenterology of 1990 [2]. An accurate nodal status is essential because there is a significant correlation between the number of nodes retrieved and survival of patients. The risk of understaging is that some patients, who would benefit from adjuvant therapy, would not be offered any [1]. Besides, a lower number of lymph nodes removed is associated with a poorer survival and, especially in rectal cancer, with a higher rates of local recurrence [3-6].

Preoperative radiotherapy decrease the number of lymph nodes yield in surgical specimen [7-10]. Many factors are associated to reduction of number of nodes after chemoradiotherapy: the immune response and fibrosis in lymph nodes exposed to radiotherapy, which results in a difficult identification of nodes in the specimen.

This study aims to observe the effect of preoperative chemoradiotherapy (CRT) on the number of lymph nodes retrieved in the specimen in patients undergoing a TME for rectal cancer.

## Methods

We observed retrospectively all the patients who had TME for rectal cancer at a single institution from July 2005 to May 2012; 111 cases are enrolled and satisfied the inclusion criteria (middle or low rectal cancer, no previous pelvic surgery or radiotherapy, R0-surgery, no conversion from laparoscopy to laparotomy). The operations performed by one of five surgeons who had experience in colorectal surgery (more than 20 colorectal resection/year). All the patients had TME surgical approach.

All the patients had preoperative staging with chest and abdomen CT scans, pelvic MRI and endorectal ultrasound. Patients with stage I disease or who rejected neoadjuvant therapy had primary surgery, patients with Stage IV disease were excluded from the study. Patients with stage II or III disease received neoadjuvant CRT (45 Gy in 25 fractions over a 5-week period with a combination of capecitabine 825 mg bid uninterrupted for 42 days). Eight weeks after finishing the CRT, the patients underwent surgery after restaging by MRI study and endorectal ultrasound. The type of surgery depended on the level of the tumour. We performed a TME procedure with Miles’ operation if was not possible to preserve the sphincter; otherwise, low anterior resection (LAR) was done. High-ligation of the inferior mesenteric artery was routinely, except in "dolichosigma" with the possibility to perform a tension-free anastomosis. All resection specimen were examined by 3 dedicated pathologists according to a standardized histopatologic protocol with evaluation of pTNM category including the total number of resected nodes and the number of positive nodes. All lymph nodes present must be examined histologically; nodal examination must not stop once 12 nodes have been identified. If less than 12 lymph nodes are found, consideration should be given to placing the fat into a lymph node highlighting solution [11]. Histopathologic tumor regression after neoadjuvant radiochemotherapy was classified according to Dvorak score [8].

The study have been performed at Oncologic Surgery and Implantable Systems Unit, Department of Emergency, IRCCS San Martino IST, Genoa, Italy. The study obtained the approval of ethics committee of IRCCS San Martino IST of Genoa (Italy). (protocol number 1/2013). Excel (Microsoft®) was employed to obtain the patient records.

### Statistical analysis

Correlation between numbers of lymph nodes and clinicopathological parameters was evaluated. The association was tested using the chi-squared test or Mann–Whitney U test (SPPS 14.0 for Windows, SPPS Inc. Chicago, IL). Linear regression was performed. The significant level was set to p < 0.05 for all tests.

## Results

The clinicopathological parameters are shown in Table 1. Out of the 111 patients, 63 were men and 48 women with a mean age of 67.6 yrs (range 36 – 84, SD 10.8). 79.3% of the patients were over 60 years old. All the tumours were adenocarcinoma. 35.1% of the patients with a known pT stage showed a Stage 0 tumours 6.3%, a Stage I 27.9%, a Stage II 19.8%, a Stage III 35.2%; the patients with a unknown pT stage after neoadjuvant therapy was 10.8%. Most tumours were in the lower rectum (59.5%). Sphincter sparing surgery was performed in 68.5% of the patients, APR in 31.5%. Fifty patients (45.0%) had neoadjuvant CRT. The mean time elapsed between the completion of the CRT and the surgery was 8.8 weeks (SD: +/− 1.45).

**Table 1:** Clinicopathological features of patients

**Parameters**	**Surgery only (*n*** = **61)**	**Neoadjuvant (*n*** = **50)**	***P***
Age (years):			0.44
<60	11	12	
>60	50	38	
Sex:			0.53
Male	33	30	
Female	28	20	
Tumor site:			0.04
Middle	30	15	
Lower	31	35	
Tumor grade:			0.006
G1	3	2	
G2	44	32	
G3	9	1	
Gx	5	15	
Stage:			0.06
0	3	4	
I	20	11	
II	13	19	
III	23	6	
X	2	10	
Type of surgery:			0.36
Low anterior resection	44	32	
Abdominalperineal resection	17	18	0.36
Surgical approach:			0.0009
Open	29	9	
Laparoscopy	32	41	
Surgeons:			0.03
1	18	5	
2	9	7	
3	12	14	
4	12	6	
5	10	18	
Pathologists:			0.66
1	31	29	
2	20	11	
3	5	5	
4	5	5	

The mean number of lymph nodes in this series of patients was 13.6 (SD: 7.3). The patients who received preoperative CRT presented a lower number of retrieved lymph nodes than patients who received surgery without neoadjuvant therapy (11.5, SD 6.5 vs. 15.3, SD 7.5, p = 0.006) (Table 2). 37.4% of patients who received neoadjuvant therapy had 12 or more removed lymph nodes, while 63.6% of patients not undergone a preoperative therapy had 12 or more nodes yield (p 0.05).

**Table 2:** Factors influencing lymph node yield (univariate analysis)

**Parameters**	**Lymph node**	**SD**	***P***
Total	13.6	7.3	
Age (years):			0.50
<60 (23)	14.5	8.1	
>60 (88)	13.3	7.1	
Sex:			0.79
Male (63)	13.7	7.1	
Female (48)	13.4	7.6	
Tumor site:			0.24
Middle (45)	14.6	7.6	
Lower (66)	12.9	7.1	
Tumor grade:			0.0003
G1 (5)	13.2	6.8	
G2 (76)	14.1	7.3	
G3 (10)	19.7	7.0	
Gx (20)	8.4	3.7	
Stage:			0.005
0 (7)	11.7	5.4	
I (31)	12	6.4	
II (22)	15.5	8.3	
III (39)	15.8	7.3	
X (12)	7.9	4.5	
Type of surgery:			0.65
Low anterior resection (76)	13.4	7.0	
Abdominalperineal resection (35)	14.0	7.08	
Surgical approach:			0.02
Open (38)	15.8	8.3	
Laparoscopy (73)	12.4	6.4	
Neoadjuvant:			0.006
No (61)	15.3	7.7	
Yes (50)	11.5	6.5	
Surgeons:			0.18
1 (23)	16.3	10.3	
2 (16)	13.6	7.3	
3 (26)	14.0	5.3	
4 (18)	13	6.7	
5 (28)	11.3	5.8	
Pathologists:			0.20
1 (60)	12.9	6.2	
2 (31)	14.0	7.6	
3 (10)	10.6	5.6	
4 (10)	16.8	8.1	

Other factors associated with a lower yield of lymph nodes was tumour grade, tumour staging and surgical approach. Regression of the data showed a significant association with only CRT (Table 3). Patient age, gender, tumour site, type of surgery, surgeons and pathologists had no impact on lymph node yield.

**Table 3:** Factors influencing therapy and lymph node yield (linear regression)

	**Variable**	**Lymph nodes**	**SD**	***P value***
	Total	13.6	7.3	
Tumor grade:				0.08
	G1 (5)	13.2	6.8	
	G2 (76)	14.1	7.3	
	G3 (10)	19.7	7.0	
	Gx (20)	8.4	3.7	
Stage:				0.46
	0 (7)	11.7	5.4	
	I (31)	12	6.4	
	II (22)	15.5	8.3	
	III (39)	15.8	7.3	
	X (12)	7.9	4.5	
Surgical approach:				0.19
	Open (38)	15.8	8.3	
	Laparoscopy (73)	12.4	6.4	
Neoadjuvant:				0.05
	No (61)	15.3	7.7	
	Yes (50)	11.5	6.5	

## Discussion

In colorectal cancer, an appropriate dissection of mesenteric lymph nodes provides an adequate staging of the tumour [1], which improves the evaluation of management and prognosis of the disease [3,4,6]. Furthermore, the number of lymph nodes removed during surgery is directly proportional to the probability of detecting disease in them [12]. On the other hand, an adequate resection of lymph nodes decreases the incidence of local recurrences [13,14] and improves the patient survival [15-17]. So far, it’s still missing a clear guide about the appropriate number of lymph nodes to be removed in rectal cancer.

In the literature, from single case series a 7 to 20 range has been proposed [6,18-21]. In 1990, the World Congress of Gastroenterology in Sydney supported a minimum of 12 lymph nodes shall be excised for assessment [2]. Similar numbers are endorsed by the NCI issued guidelines [1]. Quoting from the last edition of TNM classification of the American Joint Committee on Cancer, “It is important to obtain least 10 to 14 lymph nodes in radical colon and rectum resections in patients without neoadjuvant therapy [22].

The presence of both colon and rectal cancers in the same disease represents another important limitation of these guidelines.

To overcome this issue, in the present study we only investigate patients suffering from rectal cancer and being treated with a laparoscopic TME. The mean number of removed lymph nodes in our cohort (13.6 ±7.3) confirms results obtained in previous studies, showing that the average varies between 5 and 16 lymph nodes [8,10,15,16,23-27]. The variety of these numbers is due to different factors. First of all, it is related to the surgeon’s ability in performing an adequate TME. Then, the pathologist’s accuracy is fundamental for detecting metastatic lymph nodes [28,29]. The third factor influencing these numbers might be related to physical and anatomic differences of the patients.

The aim of our study is to evaluate the effect of chemotherapy and radiotherapy combination. The Dutch Colorectal Cancer Group, which randomly subdivides the patients to receive preoperative short radiotherapy course followed by TME surgery or TME surgery only, found out that there was a significant difference in the number of retrieved lymph nodes [9].

On average, we retrieved 7.7 lymph nodes from patients undergoing preoperative radiotherapy and 9.7 lymph nodes in surgery-only patients. Other studies on preoperative radiotherapy reached similar conclusions [8,10]. Wichmann and co-workers compared patients who had primary surgery and those who had CRT before surgery, and reported a significant difference in the number of excised lymph nodes between the two groups (19.1 for surgery vs. 13.6 for preoperative therapy, p = <0.05) 30. Another study obtained the same result (17 vs. 13, respectively) [27].

We observed a significant difference between the numbers of retrieved lymph nodes in both groups (11.6 vs. 15.6, p = 0.03). Hence, we can affirm that radiotherapy was associated with the reduction of lymph node surgical retrieval. The effect of single chemotherapy strategy is less clear. Sermier and co-workers observed that the impact of preoperative radiotherapy on yielding lymph nodes is time-dependent [26]. However, the authors observed that for 5 patients subjected to APR for recurrent anal cancer more than 5 months after the end of the radiotherapy the regression analysis was way different. In our cohort of patients, surgery was always performed after 8.8 weeks (SD: +/− 1.45) weeks from the end of the neoadjuvant therapy.

Age, sex and level of the tumour (mid or lower) do not significantly influence lymph node yield. This is partially in contrast with other studies where it is shown an higher lymph node yield in younger patients [8,23,24], and tumour mid rectal level [25]. Conversely, results from our cohort confirmed the lack of association between number of lymph nodes and male sex [8,23,25]. Also, the type of surgery (APR vs. LAR) and the surgeon and pathologist’s experience have been shown not to influence the lymph node yield. These data indicated that the codification of procedures between surgeons and pathologists might be strongly recommended for guarantee a standard high quality of management of colorectal cancer [27,30-33]. In the present study we considered also surgical approach (laparotomic or laparoscopic TME). All the 5 surgeons who participated at the operations were devoted to colorectal surgery and were not associated with a different number of retrieved lymph nodes. A less number of nodes retrieved in specimen of rectal cancer underwent at laparoscopic TME is the result of greater number of neoadjuvant therapy in this group of patients (high grade of difference between two groups - p: 0.0009-). We analyzed also the role of pathologists. In our center, where the mean number of colorectal resection is 120/year, surgeons and pathologists are specialized in the treatment of this illness, thus their role in the lymphadenectomy is codified and are not associated with a different number of retrieved nodes. In our opinion and in other studies [27] this is important to improve the quality of colorectal resection for cancer.

Finally, the pathological T stage and grade were shown to be predictive factors for the number of lymph nodes removed during surgery. These results were partially confirmed in previous studies [8,10,23,25,27]. However, the sample size is limited, the groups are heterogeneous at baseline and this aspect was not considered in univariate and multivariate analysis.

In conclusion, preoperative CRT a smaller lymph node yield is obtained after neoadjuvant CRT upon rectal cancer surgery. Other factors, for example stage and grade of the tumour, need to be taken into account when considering the surgical lymph node yield. All these factors must be collectively considered in the assessment of lymph node resection adequacy in rectal carcinoma. However, in our experience, the only factor associated with reducing of nodes retrieved in the specimen was the neoadjuvant therapy.

Is this the future of a correct staging of rectal cancer? The lymphadenectomy is at present an integral part of rectal surgery and surgeons must perform it correctly to improve their results. However, in rectal cancer surgeons pay duty to radiotherapy, although absolutely necessary when indicated, in the dissection. Nevertheless for the future probably another “staging system” will be necessary, in order to take into account also biologic aspects of the tumour [34-39]. This would allow identifying patients with aggressive illness and treating them with more effective and less toxic therapies, although at the moment consensus has yet to be reached with regard to indications for adjuvant chemotherapy following neo-adjuvant chemoradiation for rectal cancer [38,40].

## Conclusions

Preoperative CRT is associated with a reduction in the yield of lymph nodes in rectal cancer surgery. Other factors, such as stage and grade of the tumour, may also affect lymph node yield. All these factors should be taken into account when evaluating the adequacy of lymph node resection in rectal carcinoma.

## Abbreviations

ANOVA: Analysis of variance; APR: Abdominalperineal resection; CRT: Chemoradiotherapy; CT: Computed tomography; LAR: Low anterior resection; MRI: Magnetic resonance imaging; pTNM: Pathological tumor-node-metastasis; TME: Total mesorectal excision.

## Competing interests

The authors have no conflict of interest to disclose.

## Authors’ contributions

AB SS, ERi, ER, VF designed and drafted the manuscript. SS, ERi, ER, VF designed and drafted the manuscript SS, AN, GZ, FM, AP, MS and AM drafted the manuscript and developed the statistical analysis LDM, RB, VF drafted the manuscript. All authors read and approved the final manuscript.
